# Radiographic knee osteoarthritis in ex-elite table tennis players

**DOI:** 10.1186/1471-2474-13-12

**Published:** 2012-02-06

**Authors:** Reza Rajabi, Gillian M Johnson, Mohammad H Alizadeh, Nazanin Meghdadi

**Affiliations:** 1 Physical Education and Sport Sciences Department, University of Tehran, Tehran, Iran; 2 Centre for Physiotherapy Research, University of Otago, Dunedin, New Zealand

## Abstract

**Background:**

Table tennis involves adoption of the semi-flexed knee and asymmetrical torsional trunk movements creating rotational torques on the knee joint which may predispose players to osteoarthritis (OA) of the knee. This study aims to compare radiographic signs of knee OA and associated functional levels in ex-elite male table tennis players and control subjects.

**Methods:**

Study participants were 22 ex-elite male table tennis players (mean age 56.64 ± 5.17 years) with 10 years of involvement at the professional level and 22 non-athletic males (mean age 55.63 ± 4.08 years) recruited from the general population. A set of three radiographs taken from each knee were evaluated by an experienced radiologist using the Kellgren and Lawrence (KL) scale (0-4) to determine radiographic levels of OA severity. The intercondylar distance was taken as a measure of lower limb angulation. Participants also completed the pain, stiffness, and physical function categories of the Western Ontario and McMaster University Osteoarthritis Index (WOMAC) 3.1 questionnaire.

**Results:**

The results showed 78.3% of the ex-elite table tennis players and 36.3% of controls had varying signs of radiographic knee OA with a significant difference in the prevalence levels of definite radiographic OA (KL scale > 2) found between the two groups (*P *≤ 0.001). Based on the WOMAC scores, 68.2% of the ex-elite table tennis players reported symptoms of knee pain compared with 27.3% of the controls (*p *= 0.02) though no significant differences were identified in the mean physical function or stiffness scores between the two groups. In terms of knee alignment, 73.7% of the ex-elite athletes and 32% of the control group had signs of altered lower limb alignment (genu varum) (*p *= 0.01). Statistical differences were found in subjects categorized as having radiographic signs of OA and altered lower limb alignment (*p *= 0.03).

**Conclusions:**

Ex-elite table tennis players were found to have increased levels of radiological signs of OA in the knee joint though this did not transpire through to altered levels of physical disability or knee stiffness in these players when compared with subjects from the general population suggesting that function in these players is not severely impacted upon.

## Background

Table tennis is a racquet sport which requires players to adopt the semi-flexed knee position up to 90 degrees or more for sustained periods of time along with abrupt asymmetrical torsional trunk movements. Such movements subject the knee to excessive rotational torques due to the fixed position of the lower limb on the ground especially during forehand and backhand loop serves [1]. Similarly, the execution of rapid lateral and antero-posterior excursion movements of the lower limb during forehand and back hand strokes in order to gain control of the ball along with high bilateral jumping exposes manoeuvres expose the knee joints to extreme loading conditions. The cumulative loading on the knee predisposes the table tennis player to overuse conditions such as jumper knee [1,2].

Most of the work to date examining injuries associated with racquet sports has focused on acute injuries rather than chronic musculoskeletal conditions with evidence from these studies that table tennis players have a lower incidence of injury compared with racquet sports players [2,3]. These investigations also serve to highlight the need to examine each racquet sport on an individual basis in order to identify their characteristic injury patterns due to the unique features of each sport [3]. In terms of injury location, table tennis is distinguished by higher levels of shoulder injuries compared with either badminton or tennis [3] with the knee joint accounting for between 5% of the total number of acute injuries experienced by table tennis players [3].

The most common chronic joint problem seen in retired and ex-elite athletes engaging in any sport with lower limb loading is osteoarthritis (OA) of the knee [4] which gives rise to signs and symptoms of joint pain, aching, tenderness, stiffness and limitation of movement [5]. Elite athletes undertake rigorous training routines over a duration of many years and tend to mirror specific loading patterns on their joints which are representative of those experienced during their respective sports [6]. To date, the majority of studies examining the prevalence rates and levels of OA severity in sports people have focused on high contact sports such as soccer [6-8] and football players [9-11]. Further study dedicated to table tennis players is needed to examine the levels of degenerative OA in these players. Such information is useful not only for informing players of the possible consequences and benefits of many dedicated years to the sport but also in helping in alerting players to the need for correct training and playing protocols [1]. According to the aim of this study was to compare the radiographs and functional levels in ex-elite table tennis players and control subjects in order to add to the body of information regarding knee OA in athletes involved in this sport.

## Methods

### Subjects

Ex-elite table tennis players who had represented Iran at the national level were recruited with the assistance of the Iranian Table Tennis Federation (ITTF) which provided a list of contact details for 52 eligible individuals. Inclusion criteria for the ex-elite table tennis players were the requirements to have participated in table tennis at the national level for a minimum duration of 10 years and, to be still playing table tennis at the non-professional level at least twice weekly. Of the ex-table tennis players who were approached, 22 agreed to participate in the study (response rate 42%).

An age-and sex-matched control group of 22 community inhabitants were recruited from the general population residing within the city of Tehran by canvassing public places such as parks and shopping malls for volunteers. For the control subjects, the entry criteria were a history of not being engaged in regular exercise or sporting activities.

Subjects from either group were excluded from the study if they had diabetes, osteoporosis, or reported a history of surgical intervention and/or lower limb fractures. Subjects with a high body mass index (BMI) greater than 30 were also excluded due to the confounding influence of obesity in OA [12]. Four ex-elite table tennis players had been excluded from entering the study on the grounds of having a BMI > 30.

Ethical approval for the study was granted from the local Human Ethics Committee with all subjects providing written informed consent prior to their participation in the study.

### Outcome measures

#### Anthropometric data

**Figure 1:**
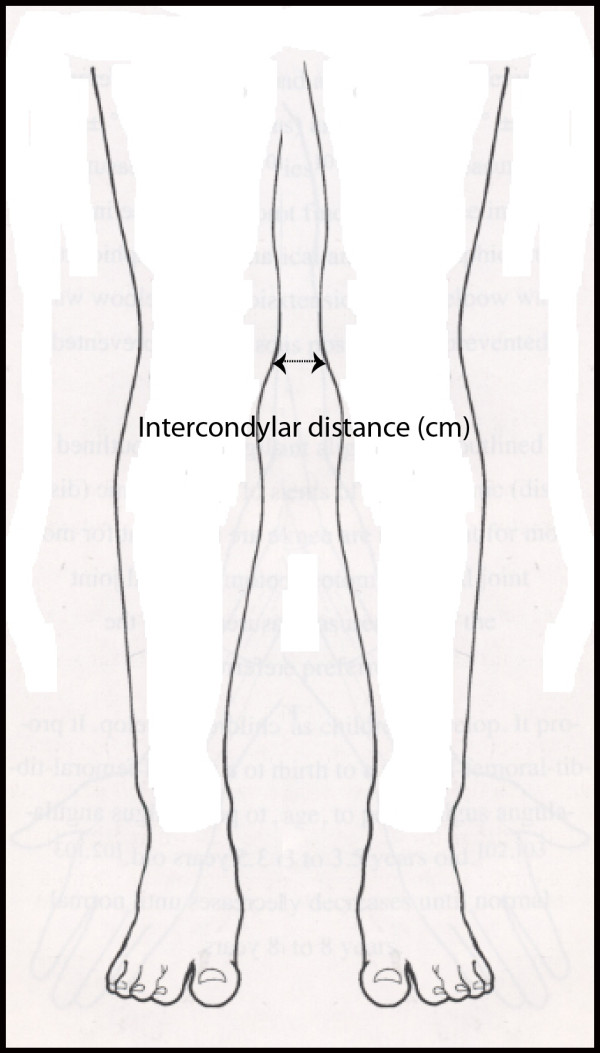
**Measurement of lower limb alignment using the intercondylar distance**.

Anthropometric details of height (cm) and weight (kg) were taken from each subject and their BMI was calculated (kg/m^2^). The intercondylar distance (cm) was measured between the medial condyles of the femur using an anthropometric caliper (Tommy 3 Bone Caliper, Rosscraft, Issaquah, USA). For this latter measurement, the subject was in barefoot standing with the feet as close together as possible and, with the weight evenly distributed on each foot according to method outlined by Sass and Hassan [13] (Figure 1). The intercondylar distance represents the degree of medial angulation of the tibia below the knee joint or genu varum ("bow legs") [14]. Subjects were then categorized as having a genu varum tendency if the intercondylar distance exceeded 5 cm.

### Smoking status

Due the possible influence of smoking status on severity of radiographic osteoarthritis in the knee joint details [15] of whether the subjects was a smoker or an ex-smoker was gathered and they were categorized smoker/ex-smoker or non-smoker accordingly.

### Radiographic grades

For each subject a bilateral set of radiographic films (antero-posterior, lateral and patello-femoral [PF] joint) was taken of both knees. Standard radiographic techniques were used with antero-posterior and lateral views taken of the tibial-femoral (TF) joint of the knee with the subject registered in the standing position and, for the PF joint view, in the sitting position, so as to optimise visualization of the respective knee joint spaces [16].

The radiographs were graded using the Kellgren and Lawrence (KL) 0-4 scale [17] using the criteria as outlined in Table 1[17]. All radiographs were graded by a single experienced radiologist who was blinded to knowledge of the subject's group status. For the purposes of analysis definite radiographic OA was defined as a KL grade of greater than 2 with the final grade assigned to a subject being the highest grade in the radiographic films of the most severely affected knee [15].

**Table 1:** Levels of radiographic knee osteoarthritis according to the Kellgren and Lawrence scale

Stage	Kellgren & Lawrence criteria	Ex-elite table tennis players (n = 22)	Control subjects (n = 22)
0	Normal	5 (22.7%)	14 (63.7%)
1	Doubtful narrowing of joint space and possible osteophytic lipping	5 (22.7%)	7 (31.7%)
2	Definite osteophytes and possible narrowing of JS	6 (27.3%)	1 (4.5%)
3	Moderate multiple osteophytes, definite narrowing of JS, some sclerosis and possible deformity of bone contour	5 (22.7%)	0
4	Large osteophytes, marked narrowing of JS, severe sclerosis, and definite deformity of bone contour	1 (4.6%)	0

JS = joint space

### Western Ontario and McMaster University Osteoarthritis Index (WOMAC)

Subjects completed the Western Ontario and McMaster University Osteoarthritis Index (WOMAC 3.1) [18]. The WOMAC is a self-administered questionnaire designed to monitor the course of osteoarthritis and the Pain, Stiffness and Physical Function categories of the questionnaire were used to evaluate symptoms and functioning. Activities in each categories were graded according to the level of difficulty using a 0-4 Likert scale which ranged from none to extreme where the response of 0 = none, 1 = mild, 2 = moderate, 3 = severe and 4 = extreme.

### Statistical analysis

Comparisons between the ex-elite table tennis players and the control group were performed using the independent student *t*-test for continuous variables and categorical data were examined using the Chi-Square Test. The criteria of definite radiographic OA (K/L > 2) and tendency for genu varum (intercondylar distance > 5) was used for group analysis. The level of statistical significance for all analyses was determined to be *p *≤ 0.05. All data were analyzed using Statistical Package of Social Science (SPSS) software for Windows (Version 17.0).

## Results

### Baseline characteristics

The baseline characteristics for the 22 ex-elite table tennis players (mean age 56.64 ± 5.17 years) with 10 years of involvement at the professional level and 22 non-athletic males age (55.63 ± 4.08 years) are detailed in Table 2. Of these variables, only the intercondylar distance was significantly different (*P *= 0.01, mean difference 1.14 cm, CI: 0.23-2.04) indicating a tendency towards genu varum in the ex-elite table tennis player group. In terms of knee alignment, 73.7% of the ex-elite athletes and 32% of the control group had signs of altered lower limb alignment (genu varum) (*p *= 0.01). There was no significant difference in numbers of smokers/ex-smokers versus non-smokers between the two groups (*P *= 0.08) (Table 2).

**Table 2:** Baseline characteristics

	Ex-elite table tennis players (n = 22)		Controls (n = 22)		
**Characteristics**	**Mean/** **percentage**	**95% CI**	**Mean/** **percentage**	**95% CI**	***P *value**

Mean age (years)	56.64	66.67-46.41	55.63	61.67-49.59	0.52
Mean height (cm)	172.63	187.07-158.19	174.00	187.03-160.97	0.53
Mean weight (kg)	76.31	92.75-59.87	77.36	91.65-63.07	0.66
Mean body mass index (kg/m^2^)	25.30	29.74-20.86	25.60	29.19-22.01	0.63
Intercondylar distance (cm)	4.96	7.97-1.95	3.78	6.95-0.81	0.01**
Smokers/ex-smokers (%)	13.6		36.4		0.08

** *P *≤ 0.01 Student *T*-test

### Radiographic knee osteoarthritis outcomes

From the radiographs and, according to the criteria of > 2 on the KL scale the prevalence levels of definite radiographic OA was 54.5% (n = 12) for the ex-elite table tennis players and 4.5% (n = 1) for the control subjects with a significant difference found between the two groups (*P *≤ 0.001). In the breakdown of frequencies detailed in Table 1 it was found that 78.3% of the ex-elite table tennis players had varying signs of radiographic OA in their knee joints and in comparison, only 36.3% of the control group had signs of OA with correspondingly less advanced radiological signs of OA according the KL scale.

### WOMAC scores

The results of the WOMAC questionnaire assessed as a group mean difference showed that 68.2% of the ex-elite table tennis players reported symptoms of knee pain compared with only 27.3% of control group (*p *= 0.02) (Table 3). However, no significant difference was identified between the two groups for either the mean physical function (*P *= 0.11) or stiffness scores (*P *= 0.22) (Table 3).

**Table 3:** WOMAC category scores for pain, stiffness and physical function

	Ex-elite table tennis players n = 22	Control subjects n = 22			
	**Mean ± SD**	**Mean ± SD**	**Mean difference**	**95% CI**	***P *value**
**WOMAC Pain**	2.81 ± 1.25	0.82 ± 1.59	2.00	0.36-3.64	*P *= 0.02*
**WOMAC Stiffness**	0.63 ± 3.47	0.27 ± 0.55	0.36	-0.23-.29	*P *= 0.22
**WOMAC Physical Function**	5.95 ± 5.72	3.48 ± 4.28	2.47	-0.60-5.55	*P *= 0.11

*P ≤ 0.05 Student *T*-test

WOMAC = Western Ontario and McMaster University Osteoarthritis Index

### Lower limb alignment

A significant relationship (*P *= 0.03) was also identified between subjects categorized according tendency to genu varum (> 5 cm) and definite signs of radiographic OA (KL grade > 2) using the Pearson's chi-square statistic indicating a relationship between the two outcome variables.

## Discussion

In this study, a significantly higher prevalence and a pattern of more advanced radiological OA in the knee joints of ex-elite table tennis players was found when compared with control group who were matched for sex, age, weight, and height. Furthermore the table tennis players reported significantly higher levels of knee pain (Table 3) and with signs of altered lower limb alignment (genu varum) when compared with the control group (Table 1). However the results also found that the physical signs of knee OA did not transpire through to increased physical disability or knee stiffness in the ex-elite table tennis group as measured by the sub-scores in the WOMAC index. As in other countries the prevalence of knee OA in Iran is high within the general population [19] and the degree of radiographic severity for knee OA in the control group in this study compared well with age specific prevalence rates in other population studies [20].

The distinguishing feature of all racket sports including that of table tennis is that the manoeuvres often occur in asymmetrical body postures onto a semi-flexed knee and it is this feature which is likely to one of the key factors in exposing the knee to increased risk for the development of OA in the ex-elite table tennis players. The combination of rapid acceleration, deceleration, jumping and landing movements seen in table tennis players [1] are common to racket sports as a whole and collectively the results of this study, serves to highlight the increased vulnerability of racket players to knee OA when playing at the professional or the elite level over a period of at least ten years.

It is also noted that in this study that the elite table tennis players tended to have an increased varum deformity of the lower limb which is strongly indicative of destruction to the medial rather than the lateral compartment of the knee [16] and this finding may be reflective of the type of loading pattern associated with table tennis playing.

The principal contributions of this paper are twofold. Firstly this study demonstrates the increased prevalence and severity of radiographic knee OA in ex-elite table tennis players at least equivalent to other groups of older athletes at the professional and elite level such as footballers [9-11] and soccer players [6-8]. Secondarily in a more positive vein, the results also suggest that continued involvement in sport as was the case in these ex-elite table tennis players tends to confer them with some protective effects against impacts on physical disability and symptoms of knee stiffness when compared with individuals from the general population.

Previous injury to a joint and occupation [6,20-26] are among the important determinants of knee OA and one of the main limitations of this study was these details were not gathered. In keeping with recommendations that when studying OA and sporting activity a control group was included [27]. However it is acknowledged the recruitment process of selected subjects from public places may have given rise to a selection bias. Although the method of measuring lower limb alignment in this study followed a well-established clinical protocol [14] no data of the reliability or validity of this approach is available and so represents an additional limitation in the study. Furthermore, although the radiographs were taken in the weight bearing position so as to optimize visibility of the TF joint space [16] no differentiation was made between the TF and PF joint involvement on the overall radiographic scores and this information would have been useful in detailing potentially characteristic patterns of OA within the knee joint in elite table tennis players. The authors also acknowledge the fact that aetiology of OA is multifactorial [28] and many other factors such as the protective effects of engaging in regular aerobic exercise may be contributing to the favourable WOMAC disability scores [29].

## Conclusions

This study reports the finding of an increased prevalence and severity of radiographic knee OA along with an increased tendency towards genu varum and reported levels of knee pain in a group of ex-elite table tennis players when compared with subjects recruited from the general population. Paradoxically no differences in joint stiffness or physical disability were found and collectively these findings imply that the negative effects of knee OA are not impacting severely on function in this group of ex-elite athletes.

## Competing interests

The authors declare that they have no competing interests.

## Authors' contributions

RR and MA conceived the study and supervised its design, execution and data analysis. RR and NM participated in the preliminary drafting of the manuscript, data collection, management and statistical analyses. GJ assisted with statistical interpretation and wrote the paper with input from RR. All authors read and approved the final manuscript.

## Pre-publication history

The pre-publication history for this paper can be accessed here:

http://www.biomedcentral.com/1471-2474/13/12/prepub

## References

[B1] Hudetz D Kondrič M, Furjan-Mandić G, Munivrana G The knee is biological transmission Proceedings of International Science Congress-Table Tennis and the Aging Population: 12-14 June 2009; Poreč 2009 Zagreb: European Table Tennis Union, Croatian Table Tennis Association, University of Zagreb; Ljubljana: University of Ljubljana 8 12

[B2] Kondrič M Furjan-Mandić G Petrinović-Zekan L Ciliga D Lees A, Cabello D, Torres G Comparison of injuries between Slovenian table tennis and badminton players Science and Racket Sports IV 2008 London: Routledge 112 117

[B3] Kondrič M Matković BR Furjan-Mandić G Hadžić Dervišević E Injuries in Racket Sports among Slovenian Players Coll Antropol 2011 35 2 413 417 21755712

[B4] Spector TD Harris PA Hart DJ Cicuttini FM Nandra D Etherington J Wolman RL Doyle DV Risk of osteoarthritis associated with long-term weight-bearing sports: a radiologic survey of the hips and knees in female ex-athletes and population controls Arthritis Rheum 1996 39 6 988 995 10.1002/art.1780390616 8651993

[B5] Sharma L Kapoor D Issa S Epidemiology of osteoarthritis: an update Curr Opin Rheumatol 2006 18 2 147 156 10.1097/01.bor.0000209426.84775.f8 16462520

[B6] Kujala UM Kettunen J Paananen H Aalto T Battie MC Impivaara O Videman T Sarna S Knee Osteoarthritis in Former Runners, Soccer Players, Weight Lifters, and Shooters Arthritis Rheum 1995 38 4 539 546 10.1002/art.1780380413 7718008

[B7] Drawer S Fuller CW Propensity for osteoarthritis and lower limb joint pain in retired professional soccer players Br J Sport Med 2001 35 6 402 408 10.1136/bjsm.35.6.402 PMC1724418 11726474

[B8] Elleuch MH Guermazi M Mezghanni M Ghroubi S Fki H Mefteh S Baklouti S Sellami S Knee osteoarthritis in 50 former top-level soccer players: a comparative study Ann Readapt Med Phys 2008 51 3 174 178 10.1016/j.annrmp.2008.01.003 18374445

[B9] Deacon A Bennell K Kiss ZS Crossley K Brukner P Osteoarthritis of the knee in retired, elite Australian Rules footballers Med J Australia 1997 166 4 187 190 9066547 10.5694/j.1326-5377.1997.tb140072.x

[B10] Golightly YM Marshall SW Callahan LF Guskiewicz K Early-Onset Arthritis in Retired National Football League Players J Phys Act Health 2009 6 5 638 643 19953841 10.1123/jpah.6.5.638

[B11] Klunder KB Rud B Hansen J Osteoarthritis of the hip and knee joint in retired football players Acta Orthop Scand 1980 51 6 925 927 10.3109/17453678008990896 7211298

[B12] Jordan JM Luta G Renner JB Linder GF Dragomir A Hochberg MC Fryer JG Self-reported functional status in osteoarthritis of the knee in a rural southern community: The role of sociogeographic factors, obesity, and knee pain Arthritis Care Res 1996 9 4 273 278 10.1002/1529-0131(199608)9:4<273::AID-ANR1790090412>3.0.CO;2-F 8997916

[B13] Sass P Hassan G Lower extremity abnormalities in children Am Fam Phys 2003 68 3 461 468 12924829

[B14] Greene WB Genu varum and genu valgum in children: differential diagnosis and guidelines for evaluation Comp Ther 1996 22 1 22 29 8654021

[B15] Sudo A Miyamoto N Horikawa K Urawa M Yamakawa T Yamada T Uchidai T Prevalence and risk factors for knee osteoarthritis in elderly Japanese men and women J Orthop Sci 2008 13 5 413 418 10.1007/s00776-008-1254-2 18843454

[B16] Ahlback S Osteoarthrosis of the knee. A radiographic investigation Acta Radiol Diagn (Stockh) 1968 277 Suppl 7 72 5706059

[B17] Kellgren JH Lawrence JS Radiological Assessment of Osteo-Arthrosis Ann Rheum Dis 1957 16 4 494 502 10.1136/ard.16.4.494 13498604 PMC1006995

[B18] Bellamy N WOMAC: A 20-year experiential review of a patient-centered self-reported health status questionnaire J Rheumatol 2002 29 12 2473 2476 12465137

[B19] Davatchi F Jamshidi AR Banihashemi AT Gholami J Forouzanfar MH Akhlaghi M Barghamdi M Noorolahzadeh E Khabazi AR Salesi M Salari AH Karimifar M Essalat-Manesh K Hajialiloo M Soroosh M Farzad F Moussavi HR Samadi F Ghaznavi K Asgharifard H Zangiabadi AH Shahram F Nadji A Akbarian M Gharibdoost F WHO-ILAR COPCORD study (Stage 1, urban study) in Iran J Rheumatol 2008 35 7 1384 1390 18464299

[B20] Felson DT Zhang Y Hannan MT Naimark A Weissman B Aliabadi P Levy D Risk factors for incident radiographic knee osteoarthritis in the elderly: the Framingham Study Arthritis Rheum 1997 40 4 728 723 10.1002/art.1780400420 9125257

[B21] Hunter DJ Eckstein F Exercise and osteoarthritis J Anat 2009 214 2 197 207 10.1111/j.1469-7580.2008.01013.x 19207981 PMC2667877

[B22] Cooper C Snow S McAlindon TE Kellingray S Stuart B Coggon D Dieppe PA Risk factors for the incidence and progression of radiographic knee osteoarthritis Arthritis Rheum 2000 43 5 995 1000 10.1002/1529-0131(200005)43:5<995::AID-ANR6>3.0.CO;2-1 10817551

[B23] Rall KL McElroy GL Keats TE A Study of Long Term Effects of Football Injury to the Knee Mo Med 1964 61 435 438 14146123

[B24] Gelber AC Hochberg MC Mead LA Wang NY Wigley FM Klag MJ Joint injury in young adults and risk for subsequent knee and hip osteoarthritis Ann Intern Med 2000 133 5 321 328 10979876 10.7326/0003-4819-133-5-200009050-00007

[B25] Sutton AJ Muir KR Mockett S Fentem P A case-control study to investigate the relation between low and moderate levels of physical activity and osteoarthritis of the knee using data collected as part of the Allied Dunbar National Fitness Survey Ann Rheum Dis 2001 60 8 756 764 10.1136/ard.60.8.756 11454639 PMC1753811

[B26] Takeda H Nakagawa T Nakamura K Engebretsen L Prevention and management of knee osteoarthritis and knee cartilage injury in sports Br J Sport Med 2011 45 4 304 309 10.1136/bjsm.2010.082321 21357577

[B27] Felson DJ Epidemiology of hip and knee osteoarthritis Epidemiol Rev 1988 10 1 27 3066625 10.1093/oxfordjournals.epirev.a036019

[B28] Garstang SV Stitik TP Osteoarthritis - Epidemiology, risk factors, and pathophysiology Am J Phys Med Rehab 2006 85 11 S2 S11 10.1097/01.phm.0000245568.69434.1a 17079976

[B29] Wang BWE Ramey DR Schettler JD Hubert HB Fries JF Postponed development of disability in elderly runners - A 13-year longitudinal study Arch Intern Med 2002 162 20 2285 2294 10.1001/archinte.162.20.2285 12418943

